# Exploring the feasibility of theory synthesis: A worked example in the field of health related risk-taking

**DOI:** 10.1016/j.socscimed.2014.11.029

**Published:** 2015-01

**Authors:** Pandora Pound, Rona Campbell

**Affiliations:** School of Social and Community Medicine, University of Bristol, 39 Whatley Road, Canynge Hall, Bristol BS8 2PS, UK

**Keywords:** Theory, Synthesis, Sociology, Public health, Interventions, Risk-taking

## Abstract

The idea of synthesising theory is receiving attention within public health as part of a drive to design theoretically informed interventions. Theory synthesis is not a new idea, however, having been debated by sociologists for several decades. We consider the various methodological approaches to theory synthesis and test the feasibility of one such approach by synthesising a small number of sociological theories relevant to health related risk-taking. The synthesis consisted of three stages: (i) *synthesis preparation*, wherein parts of relevant theories were extracted and summarised; (ii) *synthesis* which involved comparing theories for points of convergence and divergence and bringing together those points that converge; and (iii) *synthesis refinement* whereby the synthesis was interrogated for further theoretical insights. Our synthesis suggests that serious and sustained risk-taking is associated with social isolation, liminality and a person's position in relation to the dominant social group. We reflect upon the methodological and philosophical issues raised by the practice of theory synthesis, concluding that it has the potential to reinvigorate theory and make it more robust and accessible for practical application.

## Highlights

• There is increasing discussion of synthesising theory within public health.
• We conducted a synthesis of theories of risk-taking, to test the method.
• The synthesis revealed serious risk-taking to be associated with liminality.
• Synthesis is feasible and makes theory more accessible for practical application.
• The process of synthesising theory raises philosophical issues, which we discuss.

## 1 Introduction

There is a growing interest in the synthesis of theory. Although academics have always brought together different theories to generate greater theoretical insights (e.g. Cockerham, 2005, Dixon and Banwell, 2009, Zimmerman, 2013), there is increasing evidence of a more systematic approach to theory synthesis (Hardeman et al., 2005, Lorenc et al., 2012, Bonell et al., 2013). The current impetus for this has its roots in an evidence-based approach to intervention design within public health (Craig et al., 2008, National Institute of Health and Clinical Effectiveness, 2007) and in a concern with the role that theory plays in the effectiveness of interventions (Glanz and Bishop, 2010, Prestwich et al., 2014). However, researchers seeking theories to inform interventions sometimes find that the sheer volume of theoretical literature can be overwhelming, that many apparently distinct theories overlap with one another and that it is seldom clear which theories are appropriate for a particular purpose (Hardeman et al., 2005, Davis et al., 2014). For those interested in the application of theory then, theory synthesis offers the possibility of collating, evaluating and combining theories for practical use.

The notion of taking a systematic approach to the synthesis of theory predates the current public health interest, however, and has been a subject of discussion within sociology since at least the 1980s, where it is commonly referred to as ‘metatheorising’. Ritzer (1990) notes that a systematic approach allows a deeper comprehension of theories as well as the possibility of evaluating, critically analysing and improving them. He suggests that metatheorizing would benefit sociology by generating new theories, better understood theories, and overarching perspectives. Confusingly, however, Ritzer outlines a very wide-ranging approach to metatheoretical activity, including within its purview three different tasks: First, metatheorizing to attain a deeper understanding of theory, which he refers to as M_u_. This is the identification of major cognitive paradigms within sociology and the study of theories, theorists, communities of theorists and the larger intellectual and social contexts of theories. Second, metatheorizing as a prelude to theory development (M_p_), which entails the study of existing theory to produce new sociological theory. Third, M_o_, which is the practice of studying theory in order to produce a metatheory that overarches some part (or all) of sociological theory.

Ritzer's first type of metatheory (M_u_) has a very broad reach and might more appropriately be called ‘metasociology’ (Fuhrman and Snizek 1990). Turner (1991), a sociologist and general theorist, comments that Ritzer's M_u_ and M_o_ approaches tend to serve mainly ‘as a basis for endless ‘discourse’’ (267). He notes that his own approach to synthesising theory comes closest to Ritzer's M_p_, and argues that the focus should be on the theories themselves rather than on theorists or paradigms (Turner, 1990, Turner, 1991). For Turner, theory synthesis involves pulling together existing theories and extracting and synthesising key aspects to produce robust theory that has relevance to the world outside sociology. He notes however, that his emphasis on the theories themselves rather than their intellectual context, often provokes accusations of naivety and lack of sophistication. Turner's insistence on focussing on the theories derives from a frustration with sociology and his sense that sociologists are more concerned with abstract, epistemological critiques than with developing coherent and useful explanations of social forces. As a result, he suggests, and because of a failure to synthesise knowledge and theory, sociology is ignored by policy makers (Turner, 1998). He argues that theory synthesis is the key to developing robust theories of practical relevance.

The idea of metatheory has also been adopted in the field of nursing, where it is interpreted in various different ways. Paterson et al. (2001) understands metatheory as a process of identifying major paradigms and relating theories to the larger sociocultural, historical and political context, thus taking Ritzer's more wide-ranging approach (M_u_). On the other hand, Whittemore and Roy (2002), finding the 'adaptation to chronic illness model' unable to encompass all aspects of the experience of diabetes mellitus, identify several concepts in the diabetes literature with potential to enhance the model and then combine these concepts with the 'adaptation to chronic illness model' to produce a new model. They describe their methodology – the expansion of a model to include additional concepts – as theory synthesis. Yet another interpretation is provided by Walker and Avant (2005), who consider theory synthesis to be the pulling together of theoretically unconnected pieces of information to construct a theory.

Clearly the terms theory synthesis and metatheory have great potential to confuse. To promote clarity it seems to us that ‘metatheory’ might be more appropriately used to refer to the study of theoretical paradigms within a discipline, that ‘theory construction’ could refer to the pulling together of information about a phenomenon of interest to create a theory, and that ‘theory synthesis’ could refer to the more tightly focused activity of comparing and weaving together specific, related theories of interest. Although Turner has in the past referred to his methodology (which will be described in more detail below) as metatheorising and also as ‘cumulative theorising’, he now also describes it as theory synthesis (Turner, 2013).

The practice of theory synthesis has been challenged on philosophical grounds. In 2003 a debate was published on the feasibility of synthesis in the field of international relations. Smith (2003) rejected what he regarded as the implicit positivist assumption of a call for synthesis, i.e. that ‘the truth’ can be found by combining disparate theories. Moravcsik (2003), however, rejected pluralism (favoured by other contributors to the debate) on the grounds that it suggested all theories are equally valid (132). Hellmann (2003) observed that synthesis simply means to form a whole by putting parts together. We agree with his conclusion: ‘Synthesis need not entail (anti-pluralistic) consensus nor imply some teleological notion of scientific progress. (…) Irrespective of whether we work on scientific or ordinary problems, we do so holistically by combining experience and intelligence in creative ways to come up with solutions to the puzzles at hand.’ (149) Turner (1985) had earlier reached a similar conclusion, advising sociologists not to let charges of positivism dissuade them from theory synthesis. Similarly sociologist Roger Sibeon (2004) observes that postmodernists tend to be opposed to theoretical synthesis, misunderstanding it as an attempt to stifle diversity and close theoretical debate. He counters that it is possible to accept theoretical pluralism at the same time as encouraging a cumulative approach to the development of sociological theory. Furthermore, he suggests that the synthesis of useful elements of theories is desirable not only within, but also across disciplines, and even across schools of thought that seem opposed.

We report here on the process of synthesising a small number of sociological theories of risk-taking. We have considered all the approaches outlined above but have chosen to follow Turner's methodology because it focuses squarely on the theories themselves. To our knowledge his methodology remains untested outside of his own use. Our aim then, is to explore the feasibility of achieving a meaningful theory synthesis using Turner's methodology and to reflect on the practical, methodological and philosophical issues it raises.

## 2 Locating the theories

The theories we used in the synthesis were identified as a result of a separate study which explored the ease of locating sociological theory for practical application (Pound et al., in press). Our field of interest was adolescent risk-taking and we searched for sociological theories with potential to throw light on this phenomenon. For that study we began by hand-searching all the abstracts of all volumes of the journals Sociology of Health and Illness (Volume 1, 1979–May 2012) and Social Science and Medicine (Volume 1, 1982–mid-June 2012). We reasoned that we would be more likely to find sociological theories in these journals than in generic journals of risk. We did not simply conduct an electronic search using the term ‘risk taking’ because we were aware that the phenomenon of risk-taking might be conceptualised in a variety of different ways and we did not want to rule out divergent ways of framing it. By searching within only two journals we undoubtedly missed some relevant publications and our focus on risk-taking may have diverted us from wider health-related activity. However, our aim was not to conduct an exhaustive search for all relevant theories but to determine the feasibility of *synthesising* theories.

Since we were specifically interested in sociological theories of risk-*taking*, we excluded sociological theories of risk and uncertainty as a feature of postmodernity (e.g. Giddens, 1990, Giddens, 1999), risk as a product of technological and scientific advancement (Beck, 1992) and sociocultural theories of the concept of risk (Douglas, 1992, Lupton, 1999a, Lupton, 1999b). As our focus was on theories we also excluded the large body of research into lay experiences and perceptions of risk-taking, although empirical papers containing relevant theory were included. Reviews of risk-taking (e.g. France, 2000) were excluded after being scanned for relevant theories. We did not use a formal definition of theory, but followed Sutton and Staw (1995) in simply proposing that theory should be about the answer to the question why and about the connections among phenomena.

**Fig. 1:**
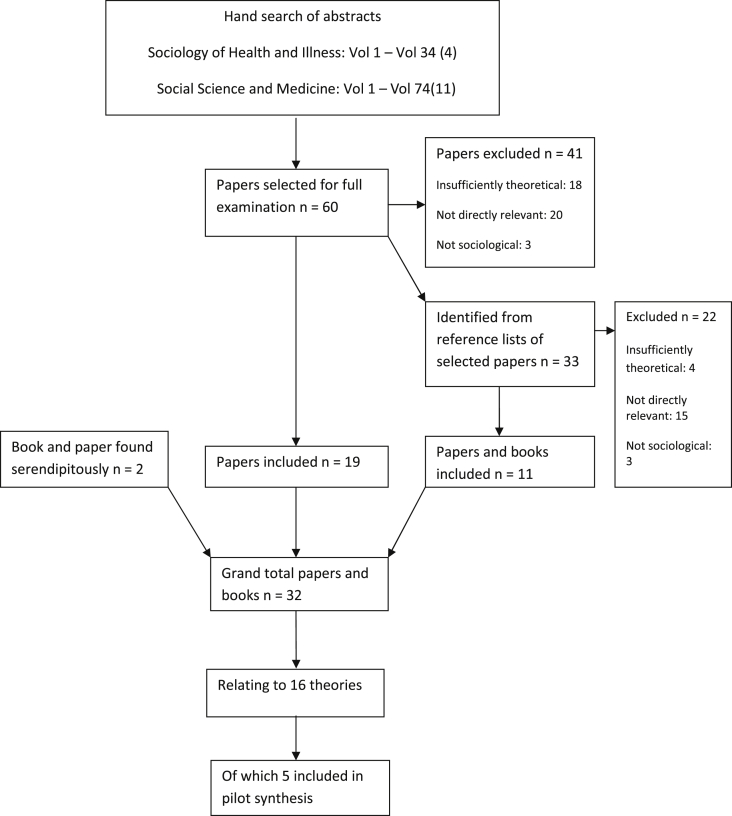
Results of search strategy to locate theories of risk-taking.

Sixty papers were identified for full examination, of which nineteen were considered relevant (Fig. 1). Promising references from the sixty papers were pursued, a process which produced a further eleven publications. In addition, two publications were found serendipitously, bringing the total to thirty two relevant publications, relating to sixteen different theories (Table 1). Five of these sixteen theories (or parts of them) related risk-taking to some aspect of social isolation and we chose these as the material for our synthesis. The theories span over a hundred years (Durkheim's ‘Suicide’ was first published in 1897 in France) and a variety of epistemological backgrounds. We felt that they had enough in common but were also sufficiently diverse – each analysing the phenomenon in markedly different ways – to provide a good test for a theory synthesis. (The five theories are identified in bold italics in Table 1).

Three of these theories were developed by sociologists (Durkheim, 1952, Becker, 1963, Factor et al., 2011), one came from social anthropology (Douglas and Calvez, 1990) while another drew upon several disciplines, including sociology, anthropology, psychology and folklore (Lightfoot, 1997). The theories are relevant to all age groups although one (Lightfoot, 1997) was developed on the basis of work with adolescents. The sorts of risk-taking activities considered by the five theorists are varied and include self-harm (suicide), sexual risk-taking, substance use, poor eating habits and low levels of physical activity.

## 3 Synthesis methodology

Turner illustrates his methodology with two examples, a synthesis of Marxist, Weberian and modern exchange theories of conflict (Turner, 1990) and a synthesis of three theories of geopolitics (Turner, 1991). For each of the syntheses he chooses theories that seem in essence similar, despite coming from different intellectual traditions. First he clarifies the concepts, models and propositions of the theories and extracts what is plausible and useful for his purposes. He attempts to state the theories simply and formally to make them easily comparable. He renders the theories more abstract (i.e. makes them pertain to all times and places rather than a specific historical or empirical context) to enable easier comparison. Turner then proceeds to synthesise a theory, or parts of a theory, with other theories. He recommends presenting the theories in tabular form to illustrate points of convergence and divergence. He breaks down the theory into propositions; those appearing on the same row address a similar dynamic, while gaps show where theories diverge or examine different processes. Finally Turner constructs an analytical model and presents it figuratively to illustrate the causal processes.

We were guided by Turner's methodology which we condensed into the following steps: 1) Synthesis preparation: the clarification of existing theories, the extraction of what is useful, plausible and relevant to the purpose of the synthesis. 2) Synthesis: making theories comparable by breaking them down into simple propositions and rendering them abstract; comparison of the theories for points of convergence and divergence; bringing together those aspects that converge. 3) Synthesis refinement: closer analysis of the product of stage 2, including an examination of causal processes, with a view to generating further theoretical insights and a more robust theory.

### 3.1 Synthesis preparation

Synthesis preparation involves extracting those parts of the theories that we are concerned with and attempting to clarify and summarise those parts. The presentation of each of the theories that follows has entailed this process of extracting, clarifying and summarising.

#### 3.1.1 Societal integration

If suicide can be regarded as an extreme form of risk-taking then Durkheim (1952) perhaps provides the first sociological theory of risk-taking. Durkheim proposed that there were three types of suicide: egoistic, altruistic and anomic, the first of which concerns us here. (Fatalistic suicide, which is sometimes considered a fourth type, is mentioned only once in a footnote.) ‘When society is strongly integrated’, wrote Durkheim, ‘it holds individuals under its control, considers them at its service and thus forbids them to dispose wilfully of themselves.’ (1952: 209) Durkheim suggested that Catholics had a lower suicide rate than Protestants because their religious community was more strongly integrated and cohesive. He concluded: ‘ … suicide varies inversely with the degree of integration of the social groups of which the individual forms a part.’ (1952: 209) In the case of egoistic suicide ‘ … the bond attaching man to life relaxes because that attaching him to society is itself slack.’ (1952: 214–215) For Durkheim then, egoistic suicide was a result of low levels of societal integration and cohesion.

#### 3.1.2 The deviant career

Becker (1963), partly on the basis of research with marijuana users, developed a theory to explain how deviance may become a way of life for some people. He suggested that for a person to progress from casual experimentation to a more sustained pattern of deviance, one of the most crucial steps is the experience of being caught and publicly labelled as deviant, since this brings about a drastic change in identity. That person is now assumed to be generally lawless and deviant in other respects and is cut off from participation in more conventional groups, perhaps becoming unemployed and drifting into marginal occupations. Becker suggests that unless the person quickly returns to the conventional community, they will continue down a path of ever increasing deviance and will be less and less subject to the impact of convention. The last step in the deviant's career is to become a member of an organised deviant group. Members of deviant groups feel a sense of common fate, Becker contends, since they are all in the same boat and face similar problems. Thus a deviant subculture grows, with a set of world views and self-justifying rationales for neutralising conventional norms. The person learns how to carry on the deviant behaviour with ease because all the problems of avoiding trouble have already been worked out and there is a stock of lore which the new member learns. Thus, suggests Becker, a person who enters an organised deviant group is highly likely to continue on that path.

#### 3.1.3 The architecture of social groups

Lightfoot (1997), who developed her theory on the basis of research with teenagers, identifies two primary clusters of risk. One is a cluster of mildly mischievous, exploratory or transitional risk-taking (e.g. experimenting with alcohol), which she regards as ‘normative’. The other is a cluster of health-compromising, destructive or pathogenic behaviours (e.g. crack cocaine addiction), which she notes are legally and culturally sanctioned as ‘deviant’. Lightfoot found that it was rare for individuals to engage in both risk clusters. She describes the latter, more serious type of risk taking as ‘marginal risk behaviour’. Her theory is that the marginality of risk coheres with the marginality of groups, i.e. those engaged in the more serious, marginal risk behaviours also belong to more marginal and isolated groups. In her view marginal risk patterns do not so much cause social isolation as *manifest* it. Lightfoot proposes that cohesion and permeability are key features to be considered. In her study, the one group characterised by a major involvement in marginal risk behaviours was also the only group with both a high degree of internal cohesion and a low degree of permeability to the wider social network. This group was more private about its risk taking and was also disengaged from the larger teenage community. By contrast, the group most active with respect to normative risk-taking was also internally cohesive but its boundaries were much more permeable and there was frequent contact with wider social networks.

#### 3.1.4 ‘Cultural theory’ of risk-taking

Douglas and Calvez (1990) argue that the self is risk-taking or risk-averse according to a predictable pattern of dealings between the person and others in the community. Their theory is that the ongoing dialogue about how to achieve the ideal community engages four different kinds of culture, each of which has a different attitude towards the self, risk-taking and the knowledge professions: 1. The ‘central community’ holds strong views on the correct norms of behaviour, is hierarchical and has developed consensus for dealing with the boundary against the outside. The authority of the established professions is accepted. The centre community is very risk-averse; when faced with a threat it will aim to consolidate the community and exclude all outsiders and repress all deviants. 2. The ‘dissenting enclaves’ protest against the central community which has rejected their principles. These enclaves espouse equality, reject the knowledge base and authority of the central community and suspect professionals. They may deride the culture of safety. 3. The ‘entrepreneurial individualists’ are highly idiosyncratic regarding health and diet but are generally risk-takers. 4. The ‘isolates’ find their activities and autonomy restricted by the other cultural types. They tend to be eccentric, which reinforces their isolation. Being isolated there is no one to challenge their ideas; they are loners who expect conspiracy and reject interference. Isolates are idiosyncratic or fatalistic in their attitude to risk. Many are explicit risk-takers in that they may be drug users and/or prostitutes. Each of the four cultures has a relationship with the centre community except for the isolates (of particular interest to this synthesis), whom the centre community expels to its margins.

#### 3.1.5 Social resistance

The thrust of Factor et al.'s (2011) theory is that non-dominant minority groups (NDMGs) tend to have greater involvement in high-risk behaviours (e.g. smoking, alcohol and drug use, poor diet, low exercise) and that these behaviours represent a form of resistance, whether conscious or unconscious, to the dominant group. The authors argue that discrimination may result in NDMGs feeling a degree of alienation from, and low attachment to, the larger society. By engaging in high-risk behaviours NDMGs are able to express their defiance of the dominant group and signal the limits of its power. Since large-scale opportunities for public resistance are few, everyday acts of resistance are more common and may act as a safety valve, enabling NDMGs to express their dissatisfaction with their status while avoiding direct negative consequences. Furthermore, argue Factor et al., NDMGs may develop a collective identity in opposition to the dominant group and may feel pressure to resist the attitudes and behaviours of the dominant group. So if healthy behaviours are associated with the dominant group, NDMGs may engage in them at the risk of hostility from their peers. The authors suggest that the power relations within society encourage members of NDMGs to actively engage in every day resistance activities which may include unhealthy behaviours.

### 3.2 Synthesis

The process of conducting a synthesis involves ‘immersion’ in the theories, allowing an opportunity to explore their meanings and possibilities in greater depth. In its careful, step by step approach it is similar to some of the activities undertaken in qualitative synthesis, particularly the process of reciprocal translation (Noblit and Hare, 1988), in which concepts are systematically compared and translated into one another. However, it is not exactly like this, since theories are broader in scope, less detailed and more abstract than qualitative findings.

#### 3.2.1 Comparison of theories for points of convergence and divergence

The theories were compared with each other and points of convergence and divergence were noted (Table 2). To enable this comparison, the theories were broken down into simple propositions and rendered abstract. For example, a proposition belonging to Becker's theory is: ‘Sustained deviant behaviour is more likely if a person is excluded from society’ (Row 2).

Regarding the causes of isolation (Row 1), for Durkheim it is because the bond attaching people to society is too slack. Becker and Factor et al. argue that it is caused by the actions and reactions of conventional society. By publicly labelling deviants, Becker argues, society effectively excludes them from conventional networks, thereby increasing social isolation. Factor et al. argue that NDMGs are alienated from wider society, possibly through discrimination. Douglas and Calvez note that some ‘isolates’ may have been expelled to the margins by the ‘centre community’ but the suggestion is that some detach themselves voluntarily. Lightfoot does not specify why ‘marginals’ are socially isolated, but the suggestion is that it is voluntary.

Durkheim, Becker, Lightfoot and Douglas and Calvez all associate *serious or persistent* forms of risk-taking with social isolation (Row 2). Durkheim argues that suicide is the result of society's failure to integrate individuals. For Becker, a pattern of sustained risk-taking is more likely if a person is socially excluded, while for Lightfoot marginal (serious) risk-taking patterns manifest social isolation. Douglas and Calvez’ ‘isolates’ have a fatalistic attitude to risk and are described as explicit risk-takers in that they may include drug users or prostitutes. Factor et al.'s theory is not concerned with serious or persistent risk-taking.

Becker and Lightfoot (Row 3) suggest that membership of a subculture or marginal group is associated with more persistent or serious forms of risk-taking, possibly due to lack of exposure to conventional norms. Becker notes that deviant group members feel drawn together by their common sense of fate into a subculture, while Lightfoot notes that marginal risk-taking (her term for serious risk-taking) is associated with membership of socially marginal groups. Douglas and Calvez’ ‘isolates’ do not belong to groups but in common with Becker's and Lightfoot's ‘deviants’ and ‘marginals’ they have little connection with wider society. Factor et al. do not consider serious or persistent risk-taking. Durkheim's theory cannot be synthesised here.

In terms of the nature of these groups (Row 4), Becker observes that deviant subcultures are internally strongly cohesive and have few links with conventional society. Similarly, Lightfoot suggest that marginal groups have high internal group cohesion and low permeability to wider social networks. Factor et al.'s NDMGs do not appear to have group identity as such, but appear to have a strong collective identity and are encouraged to resist the values of the dominant group. There may be low permeability to wider networks due to discrimination. Douglas and Calvez’ ‘isolates’ do not belong to groups. Durkheim's theory cannot be synthesised here.

Finally, Douglas and Calvez and Factor et al. suggest that isolates or NDMGs (respectively) may be in a relationship of opposition to the central community/dominant group (Row 5). Douglas and Calvez note that isolates may reject the norms and values of the centre community. Factor et al.'s theory is that risk-taking is an expression of resistance to the dominant group, illustrating dissatisfaction with inequality as well as a denoting the limits of the dominant group's power. Factor et al. also observe that NDMGs may be under pressure from their peers to resist the values of the dominant group and to refrain from adopting their health practices. They argue that the underlying motivation of NDMGs is to challenge the existing social order. The other theories do not consider this aspect so cannot be synthesised here.

In summary then, the theories suggest that detachment from the dominant social group is associated with risk-taking; that serious or sustained risk-taking in particular is associated with social isolation; that serious or sustained risk-taking is associated with membership of a deviant subculture or marginal group; that such groups tend to be strongly internally cohesive yet have little permeability to wider social networks and; that risk-taking (not necessarily serious/persistent risk-taking in this case) may be associated with opposition to the dominant social group.

### 3.3 Synthesis refinement

The ‘synthesis refinement’ stage is similar to the final stages of qualitative synthesis in which the aim is to generate a novel interpretation or conceptual advancement (Pound et al., 2005, Campbell et al., 2011). Within meta-ethnography this is sometimes called a ‘lines of argument’ synthesis (Noblit and Hare, 1988) or a ‘third-order interpretation’ (Britten et al., 2002). In the same way, theory synthesis has the potential to generate an end product greater than the sum of its parts.

**Fig. 2:**
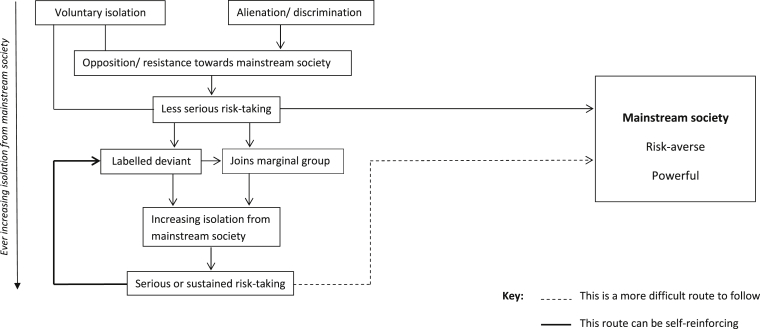
Risk-taking and its relationship to mainstream society.

We reviewed the synthesis to consider whether any further theoretical insights might be gained. We began by illustrating the causal processes suggested by the theories so far, as Turner (1990) advises, and try to bring together this next level of interpretation (Fig. 2). It is only if less serious risk-takers are labelled as deviant and/or join a marginal group that they become increasingly cut off from mainstream society and engage in serious or sustained risk-taking. This serious or sustained risk-taking is likely to reinforce the label of deviance and lead to an increasing spiral of isolation and serious risk-taking. Reintegration into mainstream society would appear to become increasingly more difficult and unlikely for those engaged in serious or sustained risk-taking. Similarly, the greater the degree of separation from mainstream society, the greater would seem the potential for serious and persistent risk-taking. Those engaged in less serious risk-taking, however, may be able to re-enter mainstream society fairly easily, perhaps as they move from childhood to adulthood, or gain status through employment. Those who gain in power have fewer reasons for resisting the dominant group, or indeed may become members of the dominant group themselves. It seems possible that reintegration into mainstream society would decrease the likelihood of engaging in serious or persistent risk-taking.

The theories referred to mainstream society in various ways. Douglas and Becker refer to the ‘centre community’, Factor et al. the ‘dominant group’ and Becker ‘conventional society’. According to Douglas and Calvez the centre community is powerful, has strong views on the correct norms of behaviour, is risk averse and when faced with a threat will try to exclude outsiders. This accords with Becker's view of conventional society. In contrast, the socially isolated groups discussed here seem markedly lacking in power, at least within conventional terms. Factor et al. and Becker are the only authors to explicitly deal with the issue of power, with their theories of resistance and deviance, respectively, but Douglas and Calvez also note that isolates are relatively powerless in relation to the centre community. Furthermore, the sort of risk-taking patterns isolates engage in (e.g. prostitution) suggest a lack of power. This raises the possibility then, that risk-taking may also be associated with powerlessness. It may be a reaction to power, i.e. signalling the limits of the dominant group's power (Factor et al.'s risk-taking as resistance), or it may be an expression of powerlessness (Douglas and Calvez’ isolates, Becker's deviants). Either way the synthesis suggests that that risk-taking is associated with powerlessness. In connection with this, many of the powerless, such as Becker's deviants and perhaps Douglas and Calvez’ isolates, seem condemned to increasing exclusion over time, with fewer and fewer possibilities to rejoin society and their power diminishing as time goes on. For this group the movement seems ever outwards, towards an existence in a liminal space on the edge of mainstream society. Consequently we propose that serious risk-taking is associated with liminality, in that serious risk-takers not only occupy a liminal space, but inhabit a liminal social status too.

In summary then, refinement of the synthesis suggests the following propositions: Serious and sustained risk-taking occurs outside the boundaries of mainstream society and is associated with powerlessness and liminality. The more detached a person becomes from mainstream society, the more likely they are to engage in serious and sustained risk-taking and the harder it will be for them to re-join mainstream society. Reintegration into mainstream society will decrease the likelihood of engaging in serious or persistent risk-taking.

## 4 Discussion

Risk-taking has previously been associated with liminality, but more commonly in the sense of pushing personal boundaries than in relation to social status. Lyng (1990) for example, conceptualised sky-diving as an exploration of edges and boundaries, coining the term ‘edgework’. Similarly Lupton and Tulloch (2002) found that participants in their qualitative study expressed risk as existing outside a defined boundary, stepping outside a comfort zone or entering no-man's land. Felix Baumgartner, who jumped to earth from the edge of space in 2012, personifies an extreme type of risk-taking in its association with liminality (http://www.redbullstratos.com/the-mission/world-record-jump/). Foucault (1994) argued that the ‘limit-experience’ may for some be a means of resisting prevailing definitions of ‘normality’ and ‘health’ and may thus represent a positive commitment to freedom and self-creation. All these examples, however, are of risk-taking as a means of exploring personal limits and usually in the context of high-risk leisure activities. Nevertheless the similarities are intriguing.

There is empirical support for the findings of our theory synthesis. A recent study from the field of criminology has identified a link between risk-taking and a marginal social status (Bengtsson, 2013) while in the field of adolescent health there is a large body of literature suggesting that rates of risk-taking are lower among children and teenagers who feel socially connected. Resnick et al. (1997), for example, found that family-connectedness and perceived school connectedness were protective against every measure of health-related risk taking except history of pregnancy. Adolescents who feel connected to their family are more likely to delay sexual initiation, report lower levels of substance use and less likely to engage in violence (Viner et al., 2012). A child's sense of belonging and connectedness to their school, a sense of neighbourhood belonging and parental involvement are all related to lower engagement in health-related risk taking (Brooks et al., 2012). Similarly a ‘whole school’ intervention that aimed to increase children's sense of attachment and connectedness reduced health-related risk taking by 25% (Patton et al., 2006).

Given the methodological nature of this paper it is not our intention to dwell here on the policy implications of our synthesis except to note that interventions may need to be fairly high upstream if they are to be effective. Wacquant (2008), for example, argues that the rise of ‘advanced marginality’ requires radical solutions such as a ‘citizen's wage’ since, increasingly, employment cannot be guaranteed to reduce poverty or marginality. Specific measures may be necessary to help reintegrate those on a downward spiral towards increasing exclusion and powerlessness. Welcoming back the deviants and the outsiders is not usually a popular measure; as Douglas and Calvez point out, the preferred approach is to exclude them beyond the bounds of the central community. Yet policies that encourage the excluded and powerless to return to society's fold are surely necessary if a cumulative cycle of exclusion and serious risk-taking is to be avoided.

### 4.1 The practice of theory synthesis

Each of the theories we considered here was valuable in its own right but we would argue that greater value has been created in their synthesis. The process enabled us to draw out the implications of each theory more fully and to produce a more robust and generalisable theory. At the beginning we simply had an association between risk-taking and social isolation but the synthesis moved us towards a refinement of this position, i.e. that it is particularly serious and sustained risk-taking that is associated with social isolation, and furthermore, that serious risk-taking is associated with a person's position in relation to the dominant social group – in other words, with liminality and powerlessness.

This preliminary work has confirmed that the methodology is feasible but further work might test its practicability on a larger scale and consider the possibility of quality appraisal of theories. Furthermore, the issue of reproducibility, namely its feasibility and desirability, needs to be addressed. For example it would seem feasible for two people to independently conduct a synthesis of the same theories, compare the results and make an assessment of reproducibility. However, theory synthesis is an interpretive approach and while the process needs to be systematic, rigorous and grounded in the theories, a high degree of reproducibility would not necessarily be expected.

In terms of using theory synthesis for practical application another issue that needs addressing is whether to use applied theories or whether to search the general literature within a discipline for candidate theories and then apply these to the issue in question (Turner, 2013). A further important question is whether theories can be synthesised across disciplines, as Sibeon (2004) suggests. This could be a very fruitful approach, although incommensurability might prove to be an obstacle. Future work might also explore how to determine which theories to include in a synthesis. At the stage of comparing the theories in our synthesis it became obvious that Durkheim's theory operated at a much broader level than the other theories and for this reason we were unable to synthesise it beyond a basic level. We should perhaps have realised this in advance but we suspect that such issues only become apparent once a synthesis is actually attempted.

In developing a methodology for theory synthesis Turner's aim was to develop robust theories for practical application. He acknowledged that his approach might be controversial because the process of synthesis does not allow full justice to be done to the theories. As he put it, 'My strategy is sacrilegious, because I advocate removing ideas from their intellectual context, throwing those away that do not seem relevant or warranted for either conceptual or empirical reasons, and using only those ideas that seem to capture the dynamic of some generic process. The goal is to *use* theories to build better ones, not to become sociological monks copying and reciting passages from the sacred texts.' (1990: 44) As noted above, his frustration derived from what he regarded as a lost opportunity for merging theory and practice (Turner, 1991). We share his frustration because some fields within public health, a discipline for which sociological theory has great relevance, tend to be dominated by psychological theories, while sociological aspects are sometimes relegated to ‘environmental influences’ (e.g. Institute of Medicine and National Research Council (2011)). Sociology has been responsible for numerous theories commonly employed within medical practice (e.g. stigma, deviance, the sick role, illness behaviour etc.) but when it comes to public health, in the rare instances in which public health interventions are informed by theory, that theory seems more likely to come from psychology (National Institute of Health and Clinical Effectiveness, 2007, Bonell et al., 2013).

It could be argued that just as there is an ethical and scientific imperative to review and synthesise empirical findings, so it is surely correct to review and synthesise bodies of theory, particularly in fields where a large body of theoretical work has accumulated (Zhao, 1991). Sibeon (2004) contends that unless a more cumulative approach to theory development is undertaken, variants of unhelpful concepts continue to be reemployed and previous ‘explanatory failures’ are repeated or compounded. Theoretical synthesis can lead to theoretical innovation, he suggests, providing Giddens' (1984) theory of structuration as an example. We argue that theory synthesis has the potential both to reinvigorate theory within a particular discipline and to render it more robust and accessible for practical application, which would be of great value in fields where theory is required to inform policy or interventions. Sociologists need to continue to develop new theories but also to revisit, review and synthesise those that already exist.

## Figures and Tables

**Table 1:** Theories identified (those synthesised identified in bold italics).

Theory/theoretical approach	Abbreviated references^a^
***Social integration***	Durkheim (1952), Willis et al. (2002), Eckersley and Dear (2002)
Deviance as collective action	Becker (1963)
***The deviant career***	Becker (1963)
Risk and development of the social self	Lightfoot (1997), Green (1997), Christensen and Mikkelsen (2008)
***Risk and the architecture of social groups***	Lightfoot (1997)
Risk and development of self-identity	Denscombe (2001)
***Cultural theory of risk-taking***	Douglas and Calvez (1990)
***Social resistance***	Factor et al. (2011), Burr (1984), Wearing et al. (1994), Peretti-Watel and Moatti (2006)
Habitus	Bourdieu (1977, 1984), Williams (1995), Lindbladh et al. (1996), Lindbladh and Lyttkens (2002), Crawshaw (2004)
Risk transition theory	Dixon and Banwell (2009)
Social practice	Frohlich et al. (2001), Frohlich et al. (2002), Delormier et al. (2009), Chan et al (2010)
Edgework	Lyng (1990), Lyng 2005, Miller (2005)
Rites of passage	Van Gennep ([1909], 1960), Robb (1986), Garrett (1996)
Situated rationality theory	Rhodes (1997)
Social action theory	Rhodes (1997)
Systems of relevance	Bloor (1995)
Total theories = 16	Total publications = 32

a Synthesised theories (in bold italics) are included in the References. See Appendix for other theories referred to here.

**Table 2:** Comparison of theories for points of convergence and divergence.

		Durkheim: Societal integration	Becker: The deviant career	Lightfoot: Architecture of social groups	Douglas and Calvez: Cultural theory of risk-taking	Factor et al.: social resistance
1	How detachment from the dominant social group is associated with risk-taking	People commit suicide because the bond attaching them to society is too slack	Society publicly labels and excludes deviants, leading to their isolation and marginalisation	Marginals may be voluntarily isolated	Some ‘isolates’ may be expelled to margins of society by centre community Some isolates may isolate themselves voluntarily	Non-dominant minority groups are alienated from wider society, possibly through discrimination, causing marginalisation
2	Serious or sustained risk-taking is associated with social isolation	Egoistic suicide is more likely in less integrated, less cohesive societies	Sustained deviant behaviour is more likely if a person is excluded from society	Marginal (i.e. serious) risk patterns manifest social isolation	‘Isolates’ have a fatalistic attitude to risk and may be explicit risk-takers, i.e. drug users and/or prostitutes	(Not concerned with serious/persistent risk-taking)
3	Serious risk-taking is associated with membership of a marginal/deviant group		Membership of an organised deviant group will encourage likelihood of sustained deviance	The marginality (i.e. seriousness) of risk coheres with the marginality of groups	*No group identity but individuals have little connection with conventional society.*	(Not concerned with serious/persistent risk-taking)
4	Nature of group		Strong internal group cohesion. Few links with conventional society.	High internal group cohesion. Low permeability to wider networks	(No group identity)	*No group identity as such but strong collective identity. Presumably low permeability to wider networks due to alienation/discrimination*
5	Risk-taking may be associated with opposition to the dominant group				‘Isolates’ may reject the norms and risk averse nature of the centre community	Non-dominant minority groups may develop collective identity in opposition to dominant group; pressure from peers not to ‘act white’. Resistance expressed through risk-taking

Italicised propositions indicate that a level of convergence exists, but that it is not strong. Bracketed statements indicate why that element of a theory cannot be synthesised. Blank cells indicate that the theory does not consider the aspect under consideration, so cannot be synthesised.
