# Supplementary material for: Exploring the feasibility of theory synthesis: A worked example in the field of health related risk-taking
Source: Soc Sci Med. 2015 Jan;124:57–65. doi: 10.1016/j.socscimed.2014.11.029 (PMC4292939; doi:10.1016/j.socscimed.2014.11.029)
Supplement: Supplementary file 1 [file mmc1.docx]

**Appendix: Publications identified in search, with those used in synthesis identified in bold italics**

***Becker, H.S. (1963). Outsiders. New York, The Free Press.***

Bloor, M. (1995). A *user’s guide to contrasting theories of HIV-related risk behaviour*, In J. Gabe (Ed.), Medicine, Health and Risk: Sociological Approaches. (pp. 19–30) Oxford, Blackwell.

Bourdieu, P. (1977). *Outline of a theory of practice*. Cambridge, Cambridge University Press.

Bourdieu, P. (1984). *Distinction: a social critique of the judgement of taste*. London, Routledge.

Burr, A. (1984). The ideologies of despair: a symbolic interpretation of punks and skinheads' usage of barbiturates. *Social Science and Medicine, 19*(9), 929-38.

Chan, C., Deave, T., Greenhalgh, T. (2010). Childhood obesity in transition zones: an analysis using structuration theory. *Sociology of Health and Illness, 32*(5), 711-29.

Christensen, P., Mikkelsen, M. (2008). Jumping off and being careful: children’s strategies of risk management in everyday life. *Sociology of Health and Illness, 30* (1), 112-30.

Crawshaw, P. (2004*). The logic of practice in the risky community: the potential of the work of Pierre Bourdieu for theorising young men’s risk-taking*. In W. Mitchell & R. Bunton. (Eds), Young People, Risk and Leisure: Constructing Identities in Everyday Life*.* (pp. 224-242) Houndsmills, Palgrave Macmillan.

Delormier, T., Frohlich, K., Potvin, L. (2009). Food and eating as social practice – understanding eating patterns as social phenomena and implications for public health. *Sociology of Health and Illness,* *31*(2), 215-28.

Denscombe, M. (2001). Uncertain identities and health-risking behaviour: the case of young people and smoking in late modernity. *British journal of Sociology, 52*(1), 157-77.

Dixon, J. , Banwell, C. (2009). Theory driven research designs for explaining behavioural health risk transitions: the case of smoking. *Social Science and Medicine, 68*, 2206-14.

***Douglas, M. , Calvez, M. (1990). The self as risk-taker: a cultural theory of contagion in relation to AIDS. Sociological Review, 38, 445-64.***

***Durkheim, E. (First published in England 1952). Suicide. London, Routledge and Kegan Paul. Translation by Spaulding J, Simpson G.***

Eckersley, R. , Dear, R. (2002) Cultural correlates of youth suicide. *Social Science and Medicine, 55*, 1891-1904.

***Factor, R. , Kawachi, I., Williams D.R. (2011). Understanding high-risk behaviour among non-dominant minorities: a social resistance framework. Social Science and Medicine, 73, 1292-1301.***

Frohlich, K., Corin, E., Potvin, L. (2001). A theoretical proposal for the relationship between context and disease. *Sociology of Health and Illness, 23*(6), 776-97.

Frohlich, K., Potvin, L., Chabot, P., Corin, E. (2002). A theoretical and empirical analysis of context: neighbourhoods, smoking and youth. *Social Science and Medicine, 54*, 1401-17.

Garrett, C.J. (1996). Recovery from anorexia nervosa: a Durkheimian interpretation. *Social Science and Medicine, 43*(10), 1489-1506.

Green, J. (1997). Risk and the construction of social identity: children’s talk about accidents. *Sociology of Health and Illness, 19*(4), 457-79.

***Lightfoot, C. (1997). The culture of adolescent risk-taking. New York, Guilford Press.***

Lindbladh, E., Hampus Lyttkens, C., Hanson, B., Ostergren, P., Isacsson, S-O., Lindgren, B. (1996). An economic and sociological interpretation of social differences in health-related behaviour: an encounter as a guide to social epidemiology. *Social Science and Medicine, 43*(12), 1817-27.

Lindbladh, E., . Lyttkens, C. (2002). Habit versus choice: the process of decision-making in health-related behaviour. *Social Science and Medicine, 55*, 451-65.

Lyng, S. (1990). Edgework: a social psychological analysis of voluntary risk-taking. *American Journal of Sociology, 94*(4), 851-86.

Lyng, S. (2005). *Edgework. The sociology of risk-taking*. (Ed) New York, Routledge.

Miller, W.J. (2005). *Adolescents on the edge: the sensual side of delinquency*. In S. Lyng (Ed.), Edgework. The Sociology of Risk-taking (pp. 153−72), New York, Routledge.

Peretti-Watel, P., Moatti, J-P. (2006). Understanding risk behaviours: how the sociology of deviance may contribute? The case of drug-taking. *Social Science and Medicine, 63*, 675-9.

Rhodes, T. (1997). Risk theory in epidemic times: sex, drugs and the social organisation of 'risk behaviour'. *Sociology of Health and Illness, 19*, 208-27.

Robb, J.H. (1986). Smoking as an anticipatory rite of passage: some sociological hypotheses on health-related behaviour. *Social Science and Medicine, 23*(6), 621-7.

Van Gennep, A [1909] (1960). *The Rites of Passage*. London, Routledge.

Wearing, B., Wearing, S., Kelly, K. (1994). Adolescent women, identity and smoking: leisure experience as resistance. *Sociology of Health and Illness, 16*(5), 626-43.

Williams, S. (1995). Theorising class, health and lifestyles: can Bourdieu help us? *Sociology of Health and Illness, 17*(5), 577-604.

Willis, L.A., Coombs, D.W., Cockerham, W.C. , Frison, S.L. (2002). Ready to die: a postmodern interpretation of the increase of African-American adolescent male suicide. *Social Science and Medicine, 55*, 907-20.
